# Retraction Note: Systematic study of cilostazol on secondary stroke prevention: a meta-analysis

**DOI:** 10.1186/s40001-015-0125-5

**Published:** 2015-03-26

**Authors:** Yining Qian, Qi Bi

**Affiliations:** Department of Neurology, Beijing Anzhen Hospital, Capital Medical University, Beijing, 100029 China

## Retraction

The Publisher and Editor regretfully retract this article [1] because the peer-review process was inappropriately influenced and compromised. As a result, the scientific integrity of the article cannot be guaranteed. A systematic and detailed investigation suggests that a third party was involved in supplying fabricated details of potential peer reviewers for a large number of manuscripts submitted to different journals. In accordance with recommendations from COPE we have retracted all affected published articles, including this one. It was not possible to determine beyond doubt that the authors of this particular article were aware of any third party attempts to manipulate peer review of their manuscript.
